# Inhibition of Growth and Induction of Apoptosis in Fibrosarcoma Cell Lines by *Echinophora platyloba* DC: In Vitro Analysis

**DOI:** 10.1155/2013/512931

**Published:** 2013-01-14

**Authors:** Fatemeh Zare Shahneh, Samira Valiyari, Abbas Azadmehr, Reza Hajiaghaee, Saeid Yaripour, Ali Bandehagh, Behzad Baradaran

**Affiliations:** ^1^Drug Applied Research Center, Tabriz University of Medical Sciences, Tabriz, Iran; ^2^Immunology Research Center, Tabriz University of Medical Sciences, Tabriz, Iran; ^3^Department of Immunology, Qazvin University of Medical Sciences, Qazvin, Iran; ^4^Pharmacognosy and Pharmaceutics, Department of Medicinal Plants Research Center, Institute of Medicinal Plants, ACECR, Karaj, Iran; ^5^Department of Food and Drug Control, School of Pharmacy, Tehran University of Medical Science, Tehran, Iran; ^6^Plant Breeding and Biotechnology Department, Faculty of Agriculture, University of Tabriz, Tabriz, Iran

## Abstract

*Echinophora platyloba* DC plant (Khousharizeh) is one of the indigenous medicinal plants which is used as a food seasoning and medicine in Iran. The objective of this study was to examine the in vitro cytotoxic activity and the mechanism of cell death of crude methanolic extracts prepared from *Echinophora platyloba* DC, on mouse fibrosarcoma cell line (WEHI-164). Cytotoxicity and viability of methanolic extract was assessed by 3-(4,5-dimethylthiazol-2-yl)-2,5-diphenyltetrazolium bromide (MTT) and dye exclusion assay. Cell death ELISA was employed to quantify the nucleosome production result from nuclear DNA fragmentation during apoptosis and determine whether the mechanism involves induction of apoptosis or necrosis. The cell death was identified as apoptosis using terminal deoxynucleotidyl transferase- (TdT-) mediated dUTP nick end labeling (TUNEL) assay. Our results demonstrated that the extract decreased cell viability, suppressed cell proliferation, and induced cell death in a time- and dose-dependent manner in WEHI-164 cells (IC50 = 196.673 ± 12.4 **μ**g/mL) when compared with a chemotherapeutic anticancer drug, Toxol. Observation proved that apoptosis was the major mechanism of cell death. So the *Echinophora platyloba* DC extract was found to time- and dose-dependently inhibit the proliferation of fibrosarcoma cell possibly via an apoptosis-dependent pathway.

## 1. Introduction

Cancer is the major cause of human's death because of high incidence and mortality. The conventional modality for cancer therapy includes surgery, chemotherapy, and radiotherapy, separately or in combination but all of these have wide range of deficiencies and side effects. These factors highlight the essential prospects for novel therapies or therapeutic combinations to improve the survival and quality of the life of cancer individuals. An effective anticancer agent should kill cancer cells without affecting abnormal-to-normal cells. Hence, the identification of new cytotoxic drug with low side effects on immune system has developed as important area in new studies of immunopharmacology. This ideal condition is feasible by inducing apoptosis in cancer cells [1]. Apoptosis (programmed cell death) is an active physiological suicide that occur normally during development and aging and as a homeostatic mechanism to maintain cell populations in tissues. Apoptosis is characterized by unique morphological and biochemical features, including cell shrinkage, membrane blebbing, chromatin condensation, and formation of apoptotic bodies [2, 3]. Maintenance of organelle integrity, condensation, and DNA fragmentation, followed by removal of dying cells by macrophage-mediated phagocytosis. Dysfunction of the apoptotic program can promote tumor initiation, progression, and treatment resistance. The deficiency of apoptosis can result in cancer, autoimmune diseases, and spreading of viral infections [4].

Traditional medicine has been used for maintaining heath, boosting immune system function, prevention, therapy, and remission of cancer [5]. Recently, extensive studies have been dedicated to the apoptosis and the role of this processes in intervention of the lethal properties of antineoplastic agents in cancer cells. Anticancer agents induce apoptosis, so that disruption of apoptotic cell death reduces treatment sensitivity. Plants have played an important role as the source of effective anticancer agents [6, 7]. Extensive varieties of natural compounds possess significant cytotoxic as well as chemopreventive activity, which act via apoptosis. Extracts of plants used in traditional medicine also have a similar property [8]. *Echinophora* is a ten-species genus of Apiaceae that contains four species native to Iran, including *E*. *orientalis*, *E*. *sibthorpiana*, *E*. *cinerea*, *and E*. *platyloba* [9]. *Echinophora platyloba* is widely used in western and central Iran as a food seasoning and edible vegetable. Local people add the plant to pickles and tomato pastes as an antifungal and antimicrobial preservative [10].

Taking into consideration, the potent antifungal and antibacterial activity suggested that the methanolic extract of *Echinophora platyloba* DC could be a potential candidate as a cytotoxic and growth inhibitory agent. The present study was undertaken to evaluate the cytotoxicity of crude methanolic extract of *E*. *platyloba* and determine the possible mechanism of cell death in malignant and nonmalignant cell lines in vitro. Cell cytotoxicity and viability was examined by the 3-(4,5-dimethylthiazol-2-yl)-2,5-diphenyltetrazolium bromide (MTT) and dye exclusion assay. The apoptosis was determined by cell death detection and specific DNA fragmentation with cell death detection (ELISA) and terminal deoxynucleotidyl transferase- (TdT-) mediated dUTP nick end labeling (TUNEL) assay.

## 2. Materials and Methods

### 2.1. Preparation of Plant Extract

*Echinophora platyloba* DC plants were collected from the west of Iran (Shahre Kord). The Department of Botany, Institute of Medicinal Plants (IMP) of Karaj, Iran, identified the plant. A voucher specimen was deposited in the herbarium of the above-mentioned entity. The aerial parts of the plant were separated, shade dried, and grinded into powder with mortar and pestle. The prepared powder was kept in tight containers protected completely from light. Extraction of methanolic extract was carried out by macerating 100 g of powdered dry plant in 500 mL of 70% methanol for 48 h at room temperature. Then, the macerated plant material was extracted with 70% methanol solvent by percolator apparatus (2-liter volume) at room temperature. The plant extract was removed from percolator, filtered through Whatman filter paper (no. 4), and dried under reduced pressure at 37°C with rotator evaporator before being added to methanol as the solvent. The methanol extract wasfiltered and concentrated using a rotary evaporator and then evaporated to dryness. Briefly, dried extracts were dissolved in dimethyl sulphoxide (DMSO) (SIGMA, USA) to get a stock solution of 10 mg/mL. The substock solution of 0.2 mg/mL was prepared by diluting 20 *μ*L of the stock solution into 980 *μ*L serum-free culture medium, RPMI 1640 and prepared at different concentrations (the percentage of DMSO in the experiment should not exceed 0.5). The stock and substock solutions were both stored at 4°C.

### 2.2. Cell Cultures

The mouse fibrosarcoma cell line (WEHI-164) and mouse nonmalignant cell line (L929) were purchased from Pasteur Institute of Iran (cell Bank). The cells were grown and maintained in a humidified incubator at 37°C and in 5% CO_2_ atmosphere. RPMI-1640 medium (SIGMA) was supplemented with 10% heat inactivated fetal calf serum (FCS), 100 units/mL penicillin, and 100 *μ*g/mL streptomycin (all from Invitrogen Gibco) was used for cell cultures. Upon reaching appropriate confluence, the cells were passaged. After being harvested from sterile T75 culture flasks (Nunc, Denmark), the cells were counted using a hemocytometer and cell viability was determined by trypan blue exclusion. Ten thousand cells from log phase cultures were seeded in 100 *μ*L of RPMI medium supplemented with 10% fetal bovine serum per well of 96-well flat-bottom culture plates (Nunc, Denmark). The cells were incubated with the different concentration of *E*. *platyloba* extract for a defined time (24, 36, and 48 hours) [11]. Proliferative response and cell death of the *E*. *platyloba* DC, extract-treated cells were determined using MTT assay, cell death ELISA, Trypan blue exclusion cell viability assay, and TUNEL assay, respectively.

### 2.3. MTT Assay

The assay detects the reduction of MTT [3-(4,5-dimethylthiazolyl)-2,5-diphenyl-tetrazolium bromide] (Sigma) (a colorimetric technique) by mitochondrial dehydrogenase to blue formazan product, which reflects the normal function of mitochondria and hence the measurement of cytotoxicity cell and viability. 1 × 10^4^ viable cells/well were plated into the 96-well tissue culture plates (Nunc Denmark), and then incubated at 37°C overnight. The next day when cells reached >80% confluence, the media were replaced with 200 mL of fresh complete medium containing 50, 100, 200, 300, 400, 500, 600, and 800 *μ*g/mL concentrations of crude extract, no extract was added to the negative control well and Toxol (Paclitaxel: plant-derived chemotherapeutic anticancer drug from *Taxus brevifolia* L.) as a control positive [12]. After 24, 36, or 48 h, the supernatants were removed and cell layers were washed with phosphate buffered saline (PBS, Invitrogen Gibco) and incubated with MTT (50 *μ*L, 0.5 mg/mL) in RPMI 1640 without FCS for 4 h in a humidified atmosphere at 37°C according to the manufacturer's protocol. The cell cultures were centrifuged at 1000 g for 5 min and the supernatants were discarded. Subsequently, 200 *μ*L of dimethyl sulfoxide (DMSO, Sigma) and 25 *μ*L Sorenson buffer were added to dissolve the formazan crystals formed. The optical density (OD) colored solution was quantified at 570 nm wavelengths by an enzyme-linked immunoabsorbent assay reader (ELISA Reader, Bio-Rad). The absorbance of untreated cells was considered as 100%. Each extract and control was assayed in triplicate in three independent experiments. The cytotoxic effects of the extracts were estimated in terms of growth inhibition percentage and expressed as IC50 which is the concentration of compound which reduces the absorbance of treated cells by 50% with reference to the control (untreated cells). We determined that IC50 values with cytotoxicity results were more than 50% at screening concentrations. Percent growth inhibition of cells exposed to treatments was calculated as follows: % Inhibition = 100 − (Test OD/Non-treated OD) × 100) [13].

### 2.4. Cell Death Detection

Cell death detection ELISA (Roche Applied Science) was used to quantify histone-complexed DNA fragments (nucleosomes) in cytoplasm of the apoptotic cells after induction of apoptosis. Briefly, after incubation with the methanolic extract (at concentrations determined by MTT assay) for 24 h, cells were pelleted and lysed. Mouse monoclonal antibodies against single-strand DNA and histones (H1, H2a, H2b, H3, and H4) specifically detected and bound mononucleosomes and oligonucleosomes derived from cells undergoing apoptosis. Biotinylated antihistone antibodies then fixed the antibody-nucleosome complexes to the streptavidin-coated microtiter plate. The anti-DNA antibodies were conjugated with a peroxidase that reacted with the substrate ABTS [2,2V-azino-di(3-ethylbenzthiazolin-sulfonate)] to form a colored product. The remaining steps were carried out according to the instructions supplied by the manufacturer. The resulting color development, which was proportional to the amount of nucleosomes captured in the antibody sandwich, was measured at 405 nm wavelength using a Benchmark microtiter plate reader (Bio-Rad). Results were expressed as the apoptotic and necrotic percentage, calculated from the ratio of absorbance of treated (apoptotic) sample to that of the untreated (control) sample [14].

### 2.5. Dye Exclusion Assay

Cellular cytotoxicity induced by the *E*. *platyloba* extract treatment was measured with trypan blue exclusion assay. Briefly, 1 × 10^4^ cells were seeded into 96-well plates and treated with or without (as control) crude extract at specified doses for 24, 36, and 48 h. After the incubation period, the cells were harvested and washed twice with PBS. The cell pellet was then resuspended with 0.5 mL PBS. Then, 20 *μ*L of suspension was mixed with equal volume of 0.4% trypan blue (Sigma, USA Merck) and was count by Neubauer haemocytometer (Weber, England) by clear-field microscopy (Nikon, Japan). Each extract and control was assayed two times in triplicate. The cells were stained with trypan blue (Merck) and live cells were enumerated. Cell counts were expressed as mean ± standard deviation (SD).

### 2.6. TUNEL Assay

To evaluate cell death by apoptosis, an in situ cell death detection kit, POD (Roche, Germany) for DNA chromatin morphologic features was used for quantification. The procedures followed to the manufacture's guidelines. Briefly, cells were cultured on glass slides and analyzed 24 hours after treatment. Cells grown on coverslips were washed twice with PBS, air dried, and fixed for 60 min in freshly prepared 4% paraformaldehyde/PBS (Sigma-Germany), pH 7.4, at room temperature. Then, the cells were washed again twice with PBS and incubated with 3% H_2_O_2_/methanol (Merck, Germany) for 10 min. Following washing with PBS, cells were permeabilized in 0.2% Triton X-100/PBS (Sigma, Germany) for 2 min at 4°C. Samples were incubated in 50 *μ*L of TUNEL reaction mixture for 2 h at 37°C in a humidified chamber and in the dark, covered with parafilm. Omission of TdT provided the negative control for the assay, and preincubation of cells with 10 *μ*g/mL DNase I in 50 mM Tris-HCl, pH 7.4, 1 mM MgCl_2_, and 1 mg/mL BSA for 10 min at room temperature to induce DNA strand breaks artificially served as positive control. Cells were washed with PBS and incubated for 30 min in a humidified chamber, at 37°C with 50 *μ*L converter-PODS (antifluorescein antibody conjugated with peroxidase). After rinsing in PBS, the samples were incubated for 10 min with 100 *μ*L DAB (Sigma, Germany) substrate in the dark. At the end, the samples were mounted and analyzed under light microscope, where the apoptotic cells could be seen as condensed shrinked dark brown cells.

### 2.7. Statistical Analysis

The data are expressed as mean ± standard deviation (SD) for at least three independent determinations in triplicate for each experimental point. The data were analyzed using IBM SPSS Statistics 20 software. For all the measurements, two-way ANOVA followed by Duncan's new multiple range test (*P* ≤ 0.05) was used to assess the statistically significance of difference between control and *E*. *platyloba* DC, treated.

## 3. Results

### 3.1. The Effects of Methanolic Extract of *Echinophora platyloba *on Inhibition and Proliferation of WEHI-164 Cells

**Figure 1:**
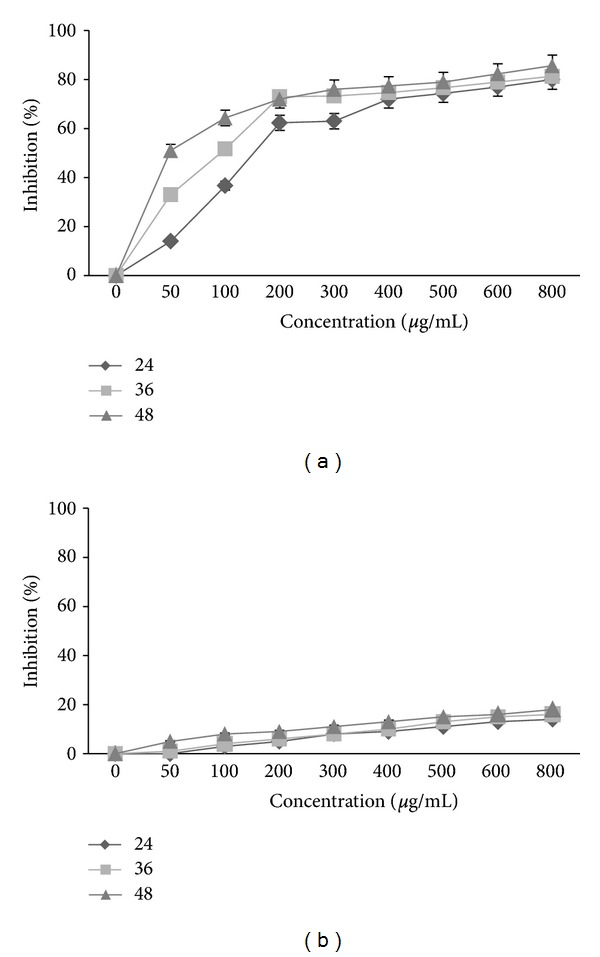
The effects of *E*. *platyloba* on proliferation of WEHI-164 cells (a) and L929 (b). Cells were incubated with increasing concentrations of *E*. *platyloba* in culture medium for 24, 36, and 48 h. The proliferative response was then assessed by MTT assay. Data presented are the mean ± SEM of three independent experiments. *P* < 0.05.

The effect of *E*. *platyloba* was studied as a time- and dose-response experiment after 24, 36, and 48 h, at concentrations of 50–800 *μ*g/mL. Proliferation of WEHI-164 cells was significantly inhibited by *E*. *platyloba* in a concentration-dependent manner in 24, 36, and 48 h, as shown in Figure 1(a) with more than 80% suppression, but there was no significant activity in L929 normal cell (IC50 > 800 *μ*g/mL) as shown in Figure 1(b) (*P* < 0.05). The concentrations producing 50% growth inhibition (IC50) of the *E*. *platyloba* extract on WEHI-164 proliferation were high potently suppressed with the IC50 value (196.673 ± 12.4 *μ*g/mL) after incubation with the *E*. *platyloba* extract.

In 24, 36, and 48 h, Dye exclusion assay was evaluated viability of WEHI-164 cells exposed to *E*. *platyloba* extract. WEHI-164 cells were compared to elucidate the cytotoxicity of both *E*. *platyloba* extract and Toxol (chemotherapeutic agent, control positive) with more than 80% in 800 *μ*g/mL and 93% in 20 *μ*g/mL (*P* < 0.01) growth suppression in 24 h (Table 1).

**Figure 2:**
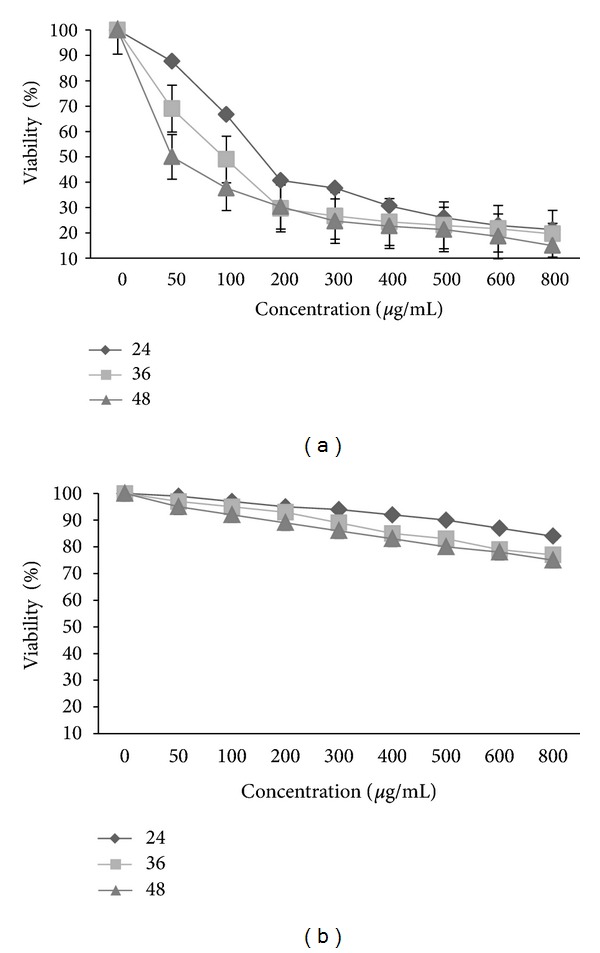
Effects of *E*. *platyloba* viability of WEHI-164 cells (a) and L929 (b). Cells were incubated with increasing concentrations of *E*. *platyloba* in culture medium for 24, 36, and 48 h. The proliferative response was then assessed by using trypan blue exclusion test. Data presented are the mean ± SEM of three independent experiments. *P* < 0.01.

Viability rate of cells exposed to *E*. *platyloba* extract at concentrations of 50 and 800 *μ*g/mL was decreased in descending form 87.3 ± 7.8% and 18.66± 2.5%, respectively (Figure 2(a)). However, L929 cells were much less susceptible to the cytotoxic effect of *E*. *platyloba* extract (Figure 2(b)).

### 3.2. The Effect of the Methanolic Extract of *Echinophora platyloba* on Cell Death of WEHI-164 Cells

**Figure 3:**
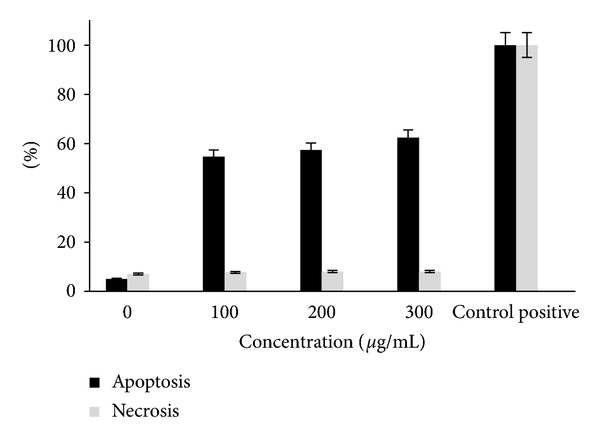
Effects of *E*. *platyloba* extract on cell viable of WEHI-164 cells. Cells were incubated with the *E*. *platyloba* 100, 200, and 300 *μ*g/mL and control positive (especial for cell death detection ELISA kit) at 24 h. The induced apoptosis (internucleosomal DNA fragmentation) was then assessed by cell death detection ELISA. Data presented are the mean ± SEM of three independent experiments.

As determined by MTT assay, *E*. *platyloba* extracts at 100, 200, and 300 *μ*g/mL in 24 h were chosen for each cell line in cell death detection ELISA. Apoptotic cell death is most likely not the only mechanism involved in downregulation of cell growth by *E*. *platyloba*. Necrosis remained at a low level (<10%) over the whole dose range (Figure 3). The possible mechanism was via induction of apoptosis, as evidenced by the significant increase in nucleosome production at 100, 200, and 300 *μ*g/mL of *E*. *platyloba* extract after incubation for 24 h but the ratio of apoptosis was constant approximately 50 ± 3%.

### 3.3. Morphological Changes Induced by the Methanolic Extract of *Echinophora platyloba *on WEHI-164 Cells

**Figure 4:**
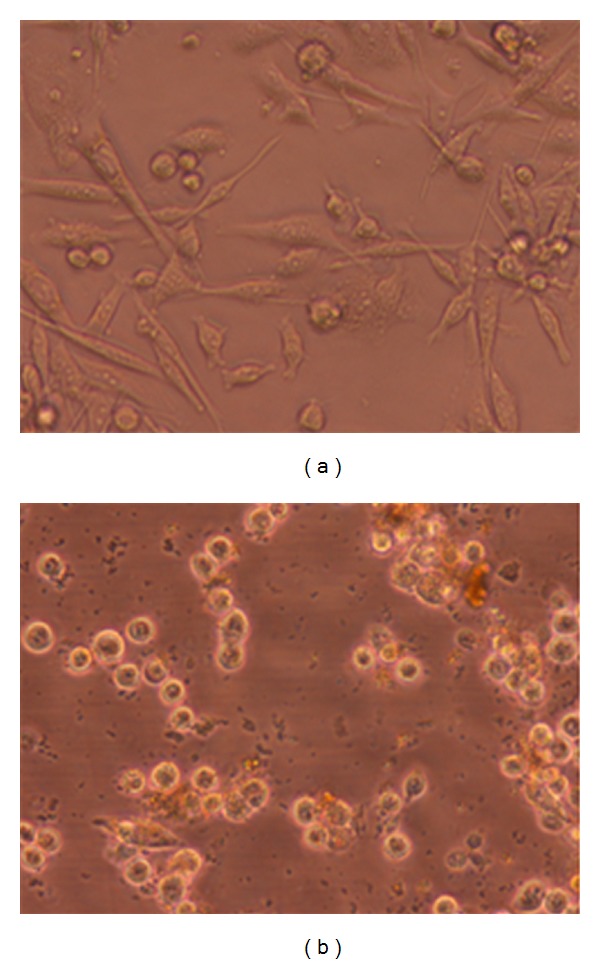
Morphological changes induced by the methanolic extract of *E*. *platyloba* on WEHI-164 cells (b) with 200 *μ*g/mL (50% growth inhibition: IC50) after 24 h treatment and comparison with untreated controls (a). Data presented are the mean ± SEM of three independent experiments.

A first approach to the mode of cell death by *E*. *platyloba* was conducted by comparison with untreated controls showing that after 24 h treatment of WEHI-164 cells with 200 *μ*g/mL (dose required for 50% inhibition), the number of cells decreased significantly and the remaining cells reduced in size with chromatin clumping (Figure 4(b)). Untreated WEHI-164 cells with normal morphology were seen in the negative control group (Figure 4(a)).

### 3.4. Quantification of Apoptosis by TUNEL Assay

**Figure 5:**
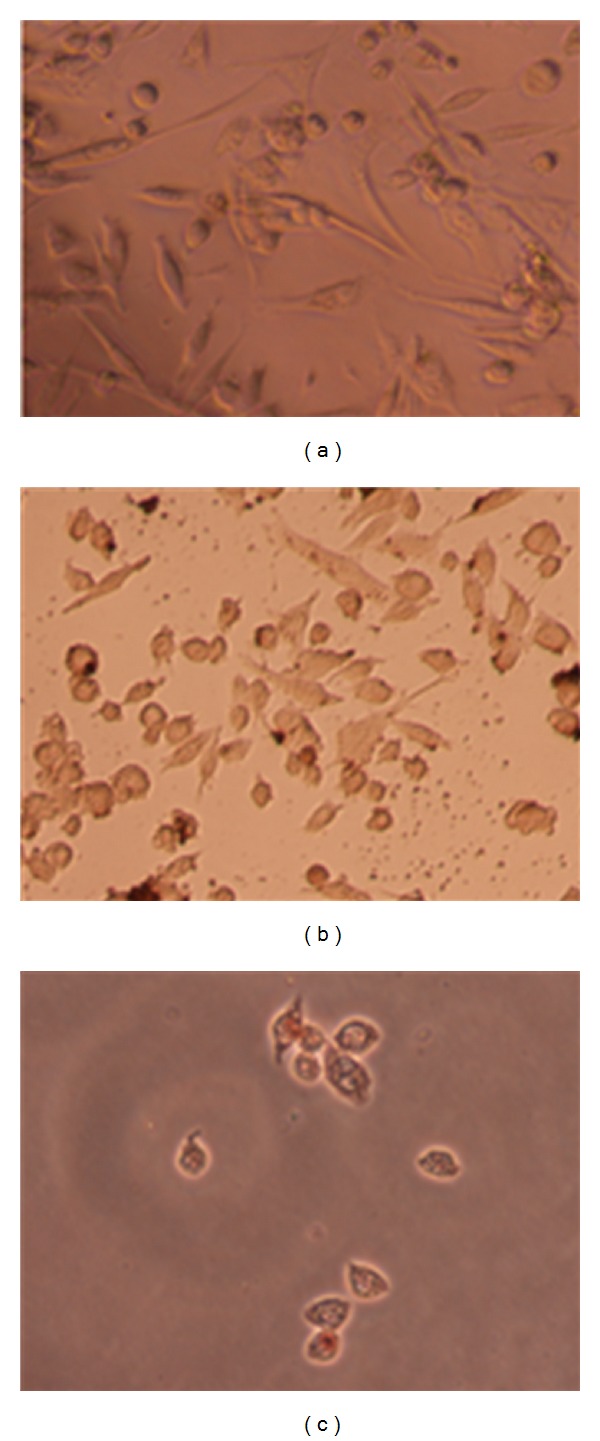
Nuclei morphological changes during *E*. *platyloba* induced apoptosis in WEHI-164 cells detected by TUNEL assay. For WEHI-164 cells, (a) shows negative control (without treatment) and (b) and (c) treated with extract (200 *μ*g: IC50) for 24 h (*n*: 3). (b) and (c) indicate representative apoptotic cells with nuclei morphological changes. Data presented are the mean ± SEM of three independent experiments. (a)–(c): 200× magnification.

TUNEL assay was done to test whether *E*. *platyloba* extract induced the decrease of cell viability and cytotoxicity contributes to apoptotic or necrosis death in WEHI-164 cell lines in vitro. It was found that the cells treated with extract (200 *μ*g/mL) for 24 h exhibited apoptotic body formation (Figure 5). Typical morphological features of apoptotic cells, with condensed and fragmented nuclei in the treatment cells with *E*. *platyloba* (Figure 5(a)), compared with homogenous nuclear chromatin in the control cells (Figure 5(b)). It was found that the WEHI-164 cells treated with *E*. *platyloba* (200 *μ*g/mL) for 24 h exhibited apoptotic body formation (Figure 5(c)).

## 4. Discussion

Apoptosis is recognized as programmed cell death, which follows in several pathological situations in multicultural organisms, and it is a form of common mechanism for cell replacement, tissue remodeling, and removal of damaged cell [2, 3]. Dysfunction of the apoptotic program can promote tumor initiation, progression, and treatment resistance. The deficiency of apoptosis can result in cancer, autoimmune diseases, and spreading of viral infections [4]. Traditional medicine has been used for maintaining heath, boosting immune system function, prevention, therapy, and remission of cancer [5]. Recently, extensive studies have been dedicated to the apoptosis and the role of this processes in intervention of the lethal properties of antineoplastic agents in cancer cells. Anticancer agents induce apoptosis, so that disruption of apoptotic cell death reduces treatment sensitivity. Plants have played a main role as the source of effective anticancer agents [6, 7]. Extensive ranges of natural compounds possess significant cytotoxic as well as chemopreventive activity, which act through apoptosis. Extracts of plants used in traditional medicine also have a similar characteristic [8].

In recent studies on medical treatment application of *E*. *platyloba*, Entezari et al. showed that the antibacterial effect on the growth of two bacteria (*Staphylococcus aureus* and *Pseudomonas aeroginosa*) of *E*. *platyloba* methanol essences and they did not observed prevention in growth of *Candida albicans, Aspergillus flavus,* and *Aspergillus niger* [15]. The current study has demonstrated the potential effect of the extracts from *E*. *platyloba* on cell growth WEHI-164 cells. MTT [3-(4,5-dimethylthiazol-2-yl)-2,5-diphenyltetrazolium bromide] assay is a common method of measuring the proliferation of cells. This assay gives an indication of intact mitochondrial and also extra mitochondrial, NADH- and NADPH-dependent redox enzyme systems. The effect of *E*. *platyloba* was studied as a time- and dose-response experiment after 24, 36, and 48 h, at concentrations of 50–800 *μ*g/mL. Proliferation of WEHI-164 cells was significantly inhibited by *E*. *platyloba* in a concentration-dependent manner in 24, 36, and 48 h, as shown in Figure 1(a) with more than 80% suppression, but there was no significant activity in L929 normal cell as shown in Figure 1(b) (*P* < 0.05). The concentrations producing 50% growth inhibition (IC50) of the E. *platyloba* extract on WEHI-164 proliferation was high potently suppressed with the IC50 value (196.673 ± 12.4, 122.977 ± 51.7 and 58.664 ± 15.2 *μ*g/mL in 24, 36, and 48 h, resp.) after incubation with the *E*. *platyloba* extract. In 24, 36, and 48 h, dye exclusion assay evaluated viability of WEHI-164 cells exposed to *E*. *platyloba* extract. Viability rate of cells exposed to *E*. *platyloba* extract at concentrations of 50 and 800 *μ*g/mL was decreased in descending form 87.3 ± 7.8% and 18.66 ± 2.5%, respectively (Figure 2(a)). However, L929 cells were much less susceptible to the cytotoxic effect of *E*. *platyloba* extract (Figure 2(b)). Reduction of cell growth can reflect either a decreased proliferation rate or an enhanced cell death by either necrosis or apoptosis or a combination of these mechanisms.

As determined by MTT assay, *E*. *platyloba* extract at 100, 200, and 300 *μ*g/mL in 24 h were chosen for each cell line in cell death detection ELISA. Apoptotic cell death is most likely not the only mechanism involved in downregulation of cell growth by *E*. *platyloba*. Necrosis remained at a low level (<10%) over the whole dose range (Figure 3). To determine whether the growth inhibitory activity of *E*. *platyloba* was related to the induction of apoptosis, the morphological character changes of WEHI-164 cells were investigated under light microscopic. In situ TUNEL assay was further carried out to confirm the cell apoptosis inducing activities of *E*. *platyloba*. TUNEL assay based on labeling of DNA strand breaks generated during apoptosis revealed that *E*. *platyloba* induces apoptosis in WEHI-164 cells. Due to degradation of DNA that resulted from the activation of Ca/Mg-dependent endonucleases in apoptotic cells, DNA cleavage occurred and led to breaking of strand within the DNA. The biotin-labeled cleavage sites were then detected by reaction with streptavidin-HRP and visualized by DAB indicating a brown color. It was found that the cells treated with extract (200 *μ*g/mL) for 24 h exhibited apoptotic body formation (Figure 4). Typical morphological features of apoptotic cells, with condensed and fragmented nuclei in the treatment cells with *E*. *platyloba* (Figure 5(a)), were compared with homogenous nuclear chromatin in the control cells (Figure 5(b)). It was found that the WEHI-164 cells treated with *E*. *platyloba* (200 *μ*g/mL) for 24 h exhibited apoptotic body formation (Figure 5(c)).

Further studies are required to assess whether cell cycle arrest or apoptosis induction contribute more to the *E*. *platyloba* extract-induced cytotoxic effect on panel cells. In order to elucidate the cytotoxic activities of *E. platyloba* extract on the growth of WEHI-164 cell line, different malignant cells may become the target cells used in our future studies. In addition, mechanistic studies on cell cycle arrest and early apoptotic events may be conducted to delineate other possible antitumor mechanisms of the *E. platyloba* extract. Besides, future in vivo antitumor studies will be performed in order to confirm these in vitro results.

## 5. Conclusion

In conclusion, the present study is the first to show cytotoxicity of *E*. *platyloba* in malignant cell lines in which apoptosis or programmed cell death plays an important role. It could provide further knowledge of mechanisms involved in this cytotoxicity. *E*. *platyloba* could be also considered as a promising chemotherapeutic agent in cancer treatment.

## Figures and Tables

**Table 1a:** (a) Toxol

Concentration (*μ*g/mL)	Growth inhibition %
20	93.78
15	82.86
10	71.69
5	63.88
0	9.3

**Table 1b:** (b) *E. platyloba*  DC

Concentration (*μ*g/mL)	Growth inhibition %
800	82.33
600	79.44
500	76.66
400	74.66
0	9.8

## Abbreviations

MTT: 3-(4,5-Dimethylthiazol-2-Yl)-2,5-diphenyltetrazolium bromide
ELISA: Enzyme-linked immunosorbent assay
TUNEL: Terminal Deoxynucleotidyl transferase- (TdT-) Mediated DUTP nick end labeling
DMSO: Dimethyl sulfoxide
FCS: Fetal calf serum.
